# Chloride Ion Transport in Reinforced Concrete Structures Considering the Barrier Effect of Reinforcing Steel

**DOI:** 10.3390/ma19061090

**Published:** 2026-03-12

**Authors:** Ying Chen, Zhimiao Ye, Yaping An, Xinhui Xiao

**Affiliations:** 1 Department of Municipal and Road-Bridge Engineering, Hunan Urban Construction College, Xiangtan 411104, China; 2 Key Laboratory of Traffic Infrastructure Security Risk Management, Changsha University of Science & Technology, Changsha 410114, China; 3 School of Civil and Environmental Engineering, Hunan University of Technology, Zhuzhou 412007, China

**Keywords:** reinforced concrete, chloride diffusion, reinforcement-induced blocking effect, meso-scale model, chloride concentration

## Abstract

A mesoscale model for chloride diffusion in reinforced concrete was established by considering the blocking effect of reinforcing steel. This model improved the accuracy of chloride concentration prediction and enhanced the reliability of durability-based service-life assessment. First, a series of chloride transport experiments under wetting–drying cycles was carried out on reinforced concrete specimens. These experiments were used to evaluate the effects of exposure condition, rebar blocking, and concrete compressive strength on chloride transport. Then, a mesoscale chloride diffusion model including the rebar-induced blocking effect was developed and validated. Finally, a sensitivity analysis of the key parameters was conducted. The results showed that, compared with concrete without longitudinal reinforcement, wetting–drying cycles had a stronger influence on reinforced concrete with longitudinal bars. The enhancing effect of wetting–drying cycles on chloride ingress was the strongest, followed by compressive strength and then the rebar-induced blocking effect, although the latter was still non-negligible. As the rebar diameter increased, the peak chloride concentration increased, and the chloride concentration around the aggregates also increased more rapidly.

## 1. Introduction

Chloride ions possess strong corrosive potential and can compromise the protective passive layer on reinforcing steel, which explains their widespread occurrence in concrete structures exposed to marine and coastal environments [1,2,3]. Moreover, reinforced concrete structures in coastal regions were widely affected by chloride ingress, which subsequently led to concrete spalling and corrosion-induced fracture of reinforcing steel. Therefore, it was necessary to investigate the chloride transport behavior in reinforced concrete structures.

In recent years, scholars have conducted extensive research on chloride transport in reinforced concrete structures. Padala et al. [4] conducted tidal drying–wetting cycles exposure experiments and measured the chloride concentration and moisture saturation at different depths in reinforced concrete members. Tešić et al. [5] performed chloride transport experiments on reinforced concrete under alternating drying–wetting cycles and analyzed the evolution of chloride concentration in the vicinity of the reinforcement. Based on field exposure tests, Chen et al. [6] compared the chloride concentration profiles under different moisture regimes, such as the tidal and splash zones. Jee et al. [7] reported that the blocking effect of reinforcement could further promote the accumulation of sulfate ions. Zacchei et al. [8] developed a multifactorial chloride ingress model for reinforced concrete under unsaturated conditions and quantified the effects of time-dependent diffusivity and exposure-related parameters on the evolution of chloride concentrations in cover concrete. Drenkard et al. [9] conducted rapid chloride migration tests on cementitious specimens and measured the resulting chloride concentration distributions after testing to clarify how sampling strategy influences the obtained chloride profiles. Kribes et al. [10] developed a multi-ion transport model for the standard chloride migration experiment by incorporating electrode processes and quantified the resulting chloride-related ion profiles to improve the accuracy of migration-based durability assessment for reinforced concrete. Although these studies investigated the spatial distribution of chloride transport in reinforced concrete or focused on chloride behavior near reinforcing bars, the influence of the rebar-induced blocking effect on chloride transport in concrete was less frequently addressed. Therefore, it was necessary to examine the effect of reinforcement-induced blocking on chloride transport in reinforced concrete structures.

Compared with physical experiments, meso-scale simulations were able to characterize the internal material structure and transport mechanisms in a highly controlled manner, significantly reducing experimental cost and duration while enhancing the depth and reproducibility of mechanistic analysis and parametric sensitivity studies [11,12,13,14,15,16]. Hu et al. [11] developed a meso-scale chloride diffusion model in which correction factors associated with temperature and relative humidity were incorporated. Pae et al. [12] simulated chloride transport pathways within concrete using a meso-scale chloride diffusion framework. Cherif et al. [13] investigated chloride transport behavior in the cement paste matrix based on thermodynamic principles. Lehner et al. [14] simulated chloride ingress-driven durability evolution in reinforced concrete by performing fully numerical service life calculations with systematically varied chloride diffusion coefficient reference times to enable controlled parametric sensitivity analysis without relying on long term exposure campaigns. Szweda et al. [15] simulated chloride migration-based durability indicators for multiple concrete mixes using a thermodynamic model of migration and compared the diffusion coefficients obtained from different standardized methods to improve the reproducibility and comparability of numerically derived transport parameters. Although these studies yielded substantial progress in meso-scale modeling of chloride transport in reinforced concrete, the blocking effect of reinforcement was less frequently considered, and the applicability of such models therefore remained open to question. Accordingly, it was necessary to establish a meso-scale chloride transport model for reinforced concrete that explicitly accounted for the rebar-induced blocking effect.

Establishing a meso-scale model for chloride diffusion in reinforced concrete that incorporated the rebar-induced blocking effect enabled an accurate investigation of chloride accumulation and flux redistribution in the vicinity of reinforcement, thereby improving the accuracy of chloride concentration predictions and the reliability of durability service-life assessments. First, a series of chloride transport experiments on reinforced concrete under drying–wetting cycles was conducted to evaluate the effects of exposure condition, reinforcement-induced blocking, and concrete compressive strength on chloride transport. Subsequently, a meso-scale chloride diffusion model accounting for the blocking effect of reinforcement was developed and validated. Finally, a mesoscale chloride diffusion model including the rebar-induced blocking effect was developed and validated. Finally, a sensitivity analysis of the key parameters was conducted.

## 2. Experiment Design

### 2.1. Material

**Figure 1:**
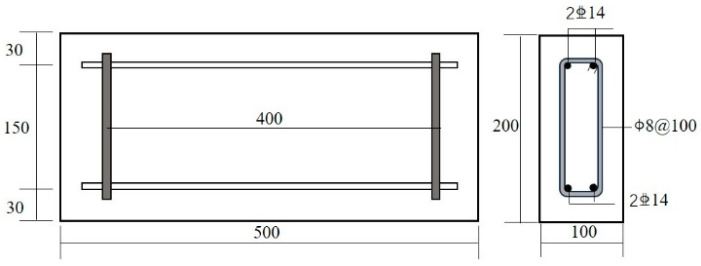
Reinforced concrete structure (Unit: mm).

To investigate the influence of chloride ions on the deterioration of reinforced concrete members and to examine the blocking effect of reinforcing steel on chloride transport, experiment beams were designed as illustrated in Figure 1. Plain concrete specimens without reinforcement were prepared as the control group for comparative analysis. Both the tension and compression zones of the test beam were reinforced with two HRB335 deformed bars serving as longitudinal load-carrying reinforcement, with a nominal diameter of 14 mm and a length of 400 mm per bar. Transverse confinement was provided by HPB300 plain round steel stirrups with a nominal diameter of 8 mm, and the concrete cover was maintained at 30 mm. The reinforced concrete specimens were produced using concrete with a target compressive strength of 50 MPa, and the corresponding mix proportions are reported in Table 1. Ordinary Portland cement with a nominal strength class of 42.5 MPa, designated as CEM I (South Cement Co., Ltd., Hangzhou, China), was employed as the primary binder. Its major oxide composition and key physical properties are provided in Table 2. The major element contents of the cement were determined by wavelength dispersive X ray fluorescence spectrometry. For XRF sample preparation, the cement powder was mixed with a binder at a mass ratio of 9:1. The results were reported in terms of the corresponding oxide contents. Pressed pellets with a diameter of 32 mm were prepared. To improve analytical accuracy, the cement was ground to a particle size below 75 μm and was handled promptly to minimize exposure to ambient air. A polycarboxylate superplasticizer (SP) (Hunan Zhongyan Building Materials Technology Co., Ltd., Changsha, China) with water reducing rate of 20% was added to improve the workability of concrete.

After casting, all concrete specimens were demolded after 24 h and then standard cured for 28 days at 20 °C and 95% relative humidity. No separate cement paste specimens were prepared. Therefore, the cement curing age refers to the specimen curing age of 28 days.

### 2.2. Chloride Penetration Experiment

This study mainly analyzes the influence of natural immersion and drying–wetting cycles on the one-dimensional transportation of chloride ions in reinforced concrete and designs the experiment conditions as shown in Table 3. There were 3 specimens in each experiment condition, totaling 24 specimens.

**Figure 2:**
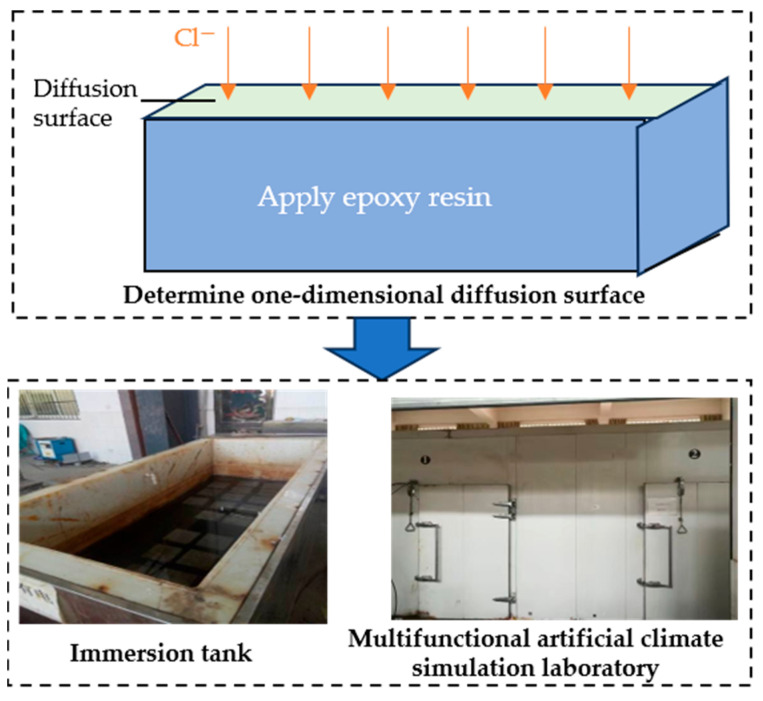
Chloride diffusion experiment of concrete specimens.

As shown in Figure 2, after curing was completed, the concrete specimens were coated with an epoxy resin on all surfaces except the designated exposure face. After the epoxy had fully hardened, the specimens were placed in an immersion tank and a multifunctional artificial climate simulation laboratory to induce chloride diffusion under natural immersion and drying–wetting cycles, respectively, using a 5% sodium chloride solution as the exposure medium. The drying–wetting cycles regime followed a 24 h cycle, consisting of 8 h of wetting and 16 h of drying. The total duration of chloride exposure was 60 days. The solution temperature for natural immersion was maintained at 20 ± 2 °C. For drying–wetting cycles, the wetting stage was conducted at 20 ± 2 °C and the drying stage was conducted at 60 ± 2 °C.

**Figure 3:**
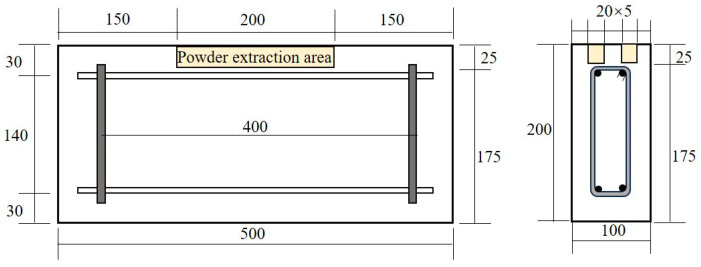
Drilling powder extraction area (Unit: mm).

After completion of the exposure time, powder samples were collected from the deteriorated surface of the concrete in accordance with the procedure reported by Kosalla et al. [17]. Sample the test piece as shown in Figure 3. Before sampling, salt crystals were removed from the exposed surface, which was then dry-cleaned. Powder was collected by grinding perpendicular to the exposed face. A 2 mm interval was used from 0 to 20 mm, and a 5 mm interval was used beyond 20 mm, following NT BUILD 443 for chloride profile sampling [18]. At least 5 g of powder was obtained from each depth to permit replicate testing. Water-soluble chloride content was measured in accordance with ASTM C1218/C1218M [19]. The thickness and number of all layers were fully recorded. The same procedure was used for all specimens to ensure comparable profile fitting and diffusion back-analysis.

To limit cross-contamination, residual powder was removed after each grinding step with a blower and a soft brush. The grinding chamber and collection area were then cleaned with deionized water. Before the next layer was sampled, the grinder was run for 2 to 3 s without collection, and the initial powder was discarded to reduce tailing from the previous layer. Blank tests were also performed, and the chloride content of the blank powder had to remain close to the detection limit to confirm cleaning effectiveness. Because chloride profiles can be influenced by extraction time and sampling position, the sampling location remained fixed throughout the test. After collection, the powder was sieved through 75 μm. For chloride extraction, a known mass of powder was mixed with deionized water at a 10:1 liquid-to-solid ratio and stirred for 30 to 60 min [20]. Free chloride content was then determined by an ion-selective electrode method after filtration, acidification, and titration with standardized AgNO_3_. Blank, dilution, and aliquot corrections were applied to calculate chloride content at each depth. For each chloride concentration category, the reported value was taken as the mean of three measurements.

### 2.3. Meso-Model of Reinforced Concrete Structure

#### 2.3.1. Chloride Ion Convection–Diffusion Equation

The chloride ion convection–diffusion equation, accounting for the coupled transport of chloride ions and moisture, is expressed by Equation (1).

(1) $$\frac{\partial C\left(x,t\right)}{\partial t}=\frac{\partial }{\partial x}\left[D_{Cl}\frac{\partial C\left(x,t\right)}{\partial x}+\frac{C\left(x,t\right)}{s(x,t)}D_{co}\frac{\partial s(x,t)}{\partial x}\right]$$

where *C*(*x*,*t*) denotes the chloride concentration at depth *x* and time *t*; *x* is the exposure depth (mm); *t* is the exposure time (s); *s*(*x*,*t*) represents the degree of moisture saturation at depth *x* and time *t*; *D*_Cl_ is the chloride diffusion coefficient of concrete (m^2^/s); and *D*_co_ is the moisture diffusion coefficient (m^2^/s).

To facilitate the investigation of the rebar blocking effect on chloride transport in a mesoscale concrete model, the following assumptions are made:

(1) In the mesoscale concrete model, chloride transport within aggregates is usually assumed to be zero. Therefore, only chloride transport in the cement paste matrix and the interfacial transition zone (ITZ) is considered.

(2) Owing to the strong coupling among rebar geometric shielding, interfacial micropore connectivity, and time-varying boundary conditions under wetting–drying cycles, parameter identification is highly challenging [21,22]. Therefore, anisotropic diffusion paths are not considered, and the local moisture gradient around the rebar (reinforcement) under drying–wetting cycles is not treated as an independent near-rebar moisture field.

The chloride diffusion coefficient of concrete is primarily governed by chloride transport in the cement paste and the interfacial transition zone, whereas chloride diffusion within aggregates is neglected [23]. The chloride diffusivities of the cement paste and the interfacial transition zone are denoted as *D*_cp_ and *D*_itz_, respectively, and their calculation follows reference [11]. The moisture diffusion coefficient mainly accounts for wetting and drying states, denoted as *D*_co_w_ and *D*_co_d_, respectively, and is described by Equation (2) [24].

(2) $$\left\{\begin{array}{c} D_{co\_w}=D_{co\_w0}\left[0.025+\frac{0.975}{1+{\left(\frac{1-s}{1-s_{c}}\right)}^{6}}\right]\\ D_{co\_d}=D_{co\_d0}exp\left(6s\right) \end{array}\right.$$

where *D*_co_w0_ is the moisture diffusion coefficient in the fully dry state (m^2^/s); *D*_co_d0_ is the moisture diffusion coefficient of saturated concrete (m^2^/s); and *s*_c_ is the moisture saturation degree at which *D*_co_w0_/2 = 0.5, with *s*_c_ taken in the range of 0.75–0.98.

#### 2.3.2. Rebar-Induced Blocking Effect

The chloride diffusion coefficients in the cement paste matrix and the interfacial transition zone (ITZ) are described by Equation (3).

(3) $$\left\{\begin{array}{c} D_{cp}=\frac{2.14\times 10^{-10}\varphi^{2.75}}{\varphi^{1.75}\left(3-\varphi \right)+14.44{\left(1-\varphi \right)}^{2.75}}\\ D_{itz}=D_{cp}\left(\frac{139.434}{d_{itz}}+1\right) \end{array}\right.$$

where *D*_cp_ and *D*_itz_ are the chloride diffusion coefficients of the cement paste matrix and the interfacial transition zone (ITZ), respectively (m^2^/s); *φ* is the porosity of the cement paste matrix; and *d*_itz_ is the thickness of the interfacial transition zone (μm).

The chloride concentration in the region ahead of the reinforcement was higher than that at the same depth in plain concrete. In practical applications, the blocking effect of reinforcement should therefore be taken into account [25,26]. Accordingly, the model for the chloride diffusion coefficient in the vicinity of the reinforcement is given in Equation (3).

(4) $$D_{cp(r)}=D_{cp}\left[2.629\left(\frac{x_{1}}{d_{r}}\right)-1.7175\right]$$

where *D*_cp(r)_ is the chloride diffusion coefficient of the cement paste affected by the reinforcement; *x*_l_ is the straight-line distance from the reinforcement along the diffusion direction; and *d*_r_ is the rebar diameter in mm. Similarly, *D*_itz(r)_ = *D*_cp(r)_·(139.434/*d*_itz_ + 1).

**Figure 4:**
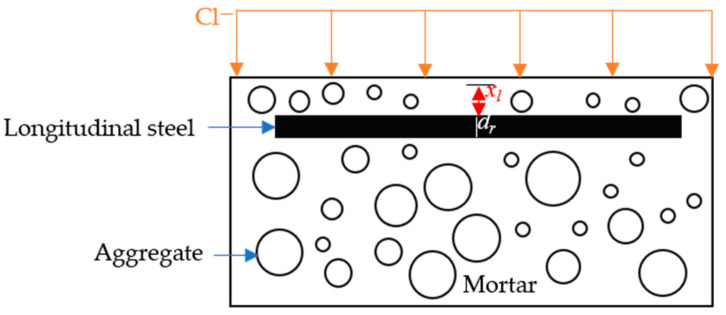
Schematic diagram of reinforcement front area.

As shown in Figure 4, *x*_l_ is taken as 16 to 20 mm. Likewise, the chloride diffusion coefficient of the interfacial transition zone influenced by the reinforcement also needs to account for the rebar-induced blocking effect.

Under drying–wetting cycles, the initial and boundary conditions for moisture and chloride transport in the concrete structure are given in Equations (5) and (6), respectively.

(1) Initial condition

(5) $$s\left(x,0\right)=s_{1},\;\;C\left(x,0\right)=C_{0}$$

(2) Boundary conditions

(6) $$\left\{\begin{array}{cc} s_{1}\left(0,t\right)=s_{2},\;\;C\left(0,t\right)=C_{s} & \left(\mathrm{Wetting}\;\mathrm{state}\right)\\ s_{2}\left(0,t\right)=s_{3},\;\;\;ng\left(D_{Cl}\cdot \nabla C+D_{co}\cdot \nabla s\cdot C\right){|}_{\left(0,t\right)}=0 & \left(\mathrm{Drying}\;\mathrm{state}\right) \end{array}\right.$$

where *s*(*x*,0) denotes the moisture saturation degree in concrete at *t* = 0; *C*(*x*,0) denotes the chloride concentration in concrete at *t* = 0; *C*(0,*t*) denotes the chloride concentration at the concrete surface (*x* = 0) at time *t*; and *s*_1_(0,*t*) and *s*_2_(0,*t*) denote the surface moisture saturation degrees (*x* = 0) under wetting and drying states, respectively. Here, *s*_1_ is the initial moisture saturation degree of concrete; *C*_0_ is the initial chloride concentration of concrete; *C_s_* is the surface chloride concentration; *s*_2_ and *s*_3_ are the surface moisture saturation degrees under the wetting state and the drying state, respectively.

#### 2.3.3. Establishment of the Model

**Figure 5:**
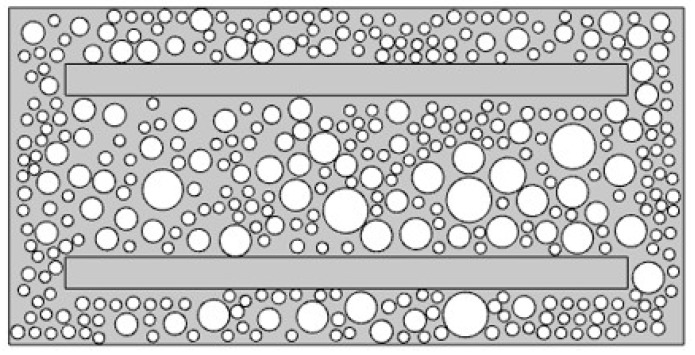
Mesoscopic model of reinforced concrete specimens.

In this study, a meso-scale reinforced concrete model was established following the approach reported in reference [27]. A two-dimensional mesoscopic model, consisting of aggregates, interfacial transition zones, and mortar as shown in Figure 5, was selected to investigate chloride diffusion.

Under the unstressed condition, the chloride concentration distribution on the midspan cross-section of reinforced concrete can represent the chloride transport behavior in the experimental model. The influence of stirrups was not considered at this stage [28]. Therefore, the midspan cross-section of the test beam was selected. The thickness of the interfacial transition zone was set to 30 μm, and the cement paste matrix and the interfacial transition zone were discretized using an unstructured quadrilateral mesh with an element size of 1 mm × 1 mm. For the reinforcement-affected region, *x_l_* was taken as 20 mm in this study. In this model, the settings of relevant parameters are shown in Table 4. The solution procedure for the meso-scale model of chloride transport in concrete under natural immersion and drying–wetting cycles conditions followed the methodology reported in reference [24].

## 3. Analysis of Experiment Results

### 3.1. Effect of the Exposure Environment

**Figure 6:**
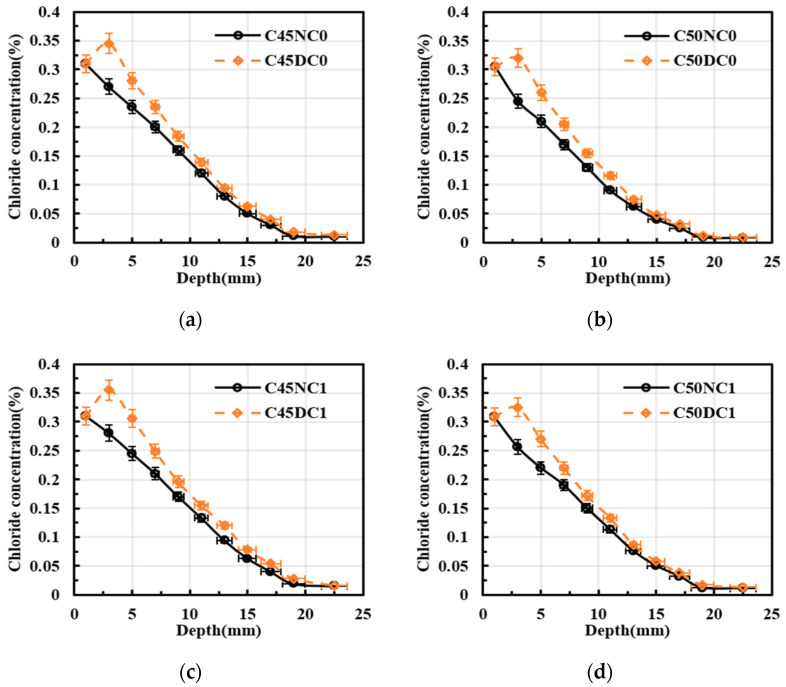
Effect of the exposure environment on chloride concentration distribution. (**a**) C45, and no longitudinal steel; (**b**) C50, and no longitudinal steel; (**c**) C45, and longitudinal steel; (**d**) C50, and longitudinal steel.

Figure 6 illustrates the effect of exposure conditions on chloride diffusion in the concrete specimens. In Figure 6a, drying–wetting cycles exerted a pronounced influence on chloride transport. The chloride concentration of C45NC0 decreased with increasing diffusion depth. In contrast, C45DC0 showed an initially increasing and subsequently decreasing trend with depth, and the maximum chloride concentration was observed at a diffusion depth of 3 mm. In addition, over the depth range of 0 to 25 mm, the mean chloride concentration of C45DC0 was 1.17 times that of C45NC0. The chloride diffusion profile in Figure 6b was consistent with that in Figure 6a. Furthermore, as shown in Figure 6c, the peak chloride concentration of C45DC1 increased by 3% relative to that of C45DC0. Over the depth range of 0 to 25 mm, the mean chloride concentration of C45DC0 was 1.22 times that of C45NC0. Compared with concrete without longitudinal reinforcement, drying–wetting cycles produced a more pronounced effect on concrete incorporating longitudinal bars. The chloride solution was rapidly drawn into the pore network during the wetting stage by capillary absorption, whereas during the drying stage moisture migration and evaporation promoted solute accumulation in the near-surface region. Repeated shrinkage and swelling cycles also induced microcracking, which further increased the connectivity of transport pathways. Consequently, the peak chloride concentration increased and the location of the concentration peak shifted inward. In contrast, under continuous immersion, chloride ingress was governed predominantly by diffusion, and the chloride profile generally decreased monotonically with depth [30,31].

### 3.2. Effect of the Rebar-Induced Blocking Effect

**Figure 7:**
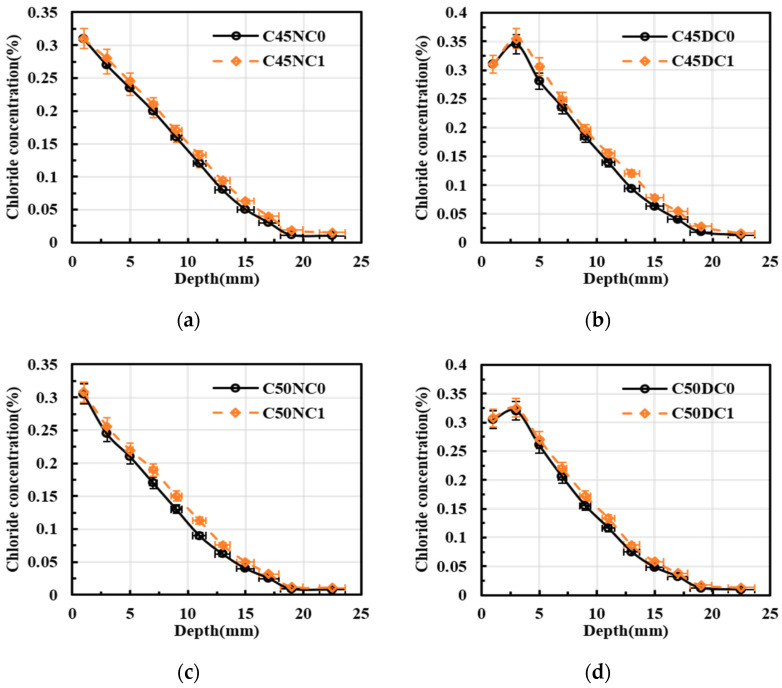
Effect of the rebar-induced blocking effect on chloride concentration distribution. (**a**) C45, and natural immersion; (**b**) C45 and drying–wetting cycles; (**c**) C50, and natural immersion; (**d**) C50, and drying–wetting cycles.

Figure 7 demonstrated the effect of the reinforcement blocking effect on chloride diffusion in the concrete specimens. In Figure 7a, over the diffusion depth range of 0 to 25 mm, the mean chloride concentration of C45NC1 increased by 7.39% relative to that of C45NC0. The blocking effect of the reinforcement became more pronounced between 9 and 17 mm, where the mean chloride concentration of C45NC1 exceeded that of C45NC0 by 13.64%. Similarly, in Figure 7b, within the 0 to 25 mm depth range, the mean chloride concentration of C45DC1 was 8.94% higher than that of C45DC0. At a diffusion depth of 13 mm, the chloride concentration of C45DC1 increased by 27.67% compared with C45DC0. The variation trends observed in Figure 7c,d were consistent with those in Figure 7a and Figure 7b, respectively. The reinforcing bar acted as a low-permeability obstacle that forced the effective diffusion paths of chloride ions to detour, thereby increasing the tortuosity of the equivalent transport channels. Local flux convergence was also formed on the chloride-exposed side of the bar, which resulted in higher chloride concentrations near the reinforcement than in plain concrete at the same depth. This effect was more pronounced within a certain depth range and was commonly associated with the cover thickness, bar diameter, and the presence of two-dimensional or bidirectional exposure boundaries. An increase in bar diameter or a reduction in cover thickness amplified chloride accumulation in the near-reinforcement region, thereby increasing the likelihood that the chloride threshold at the steel surface was reached [32,33].

### 3.3. Effect of the Compressive Strength of Concrete

**Figure 8:**
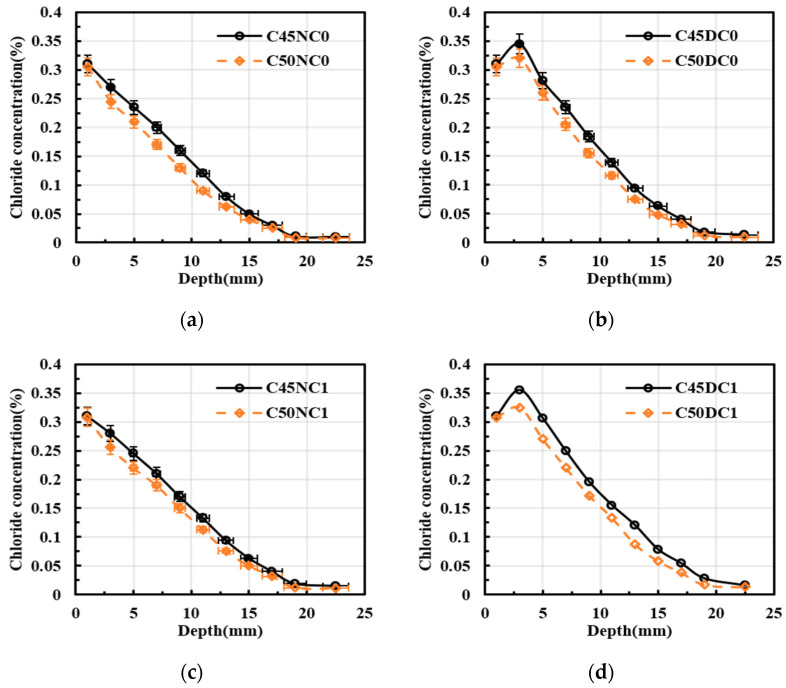
Effect of the compressive strength on chloride concentration distribution. (**a**) No longitudinal steel, and natural immersion; (**b**) No longitudinal steel and drying–wetting cycles; (**c**) Longitudinal steel, and natural immersion; (**d**) Longitudinal steel, and drying–wetting cycles.

Figure 8 revealed the influence of concrete compressive strength on chloride diffusion in the specimens. Over the diffusion depth range of 0 to 25 mm, the mean chloride concentrations of C45NC0, C45DC0, C45NC1, and C45DC1 were higher than those of C50NC0, C50DC0, C50NC1, and C50DC1 by 14.06%, 12.04%, 11.78%, and 14.14%, respectively. These results indicated that a reduction in compressive strength suppressed chloride transport in concrete. Overall, drying–wetting cycles provided the strongest enhancement of chloride ingress, followed by compressive strength, and then the rebar-induced blocking effect. Nevertheless, the blocking effect of reinforcement remained non-negligible. A higher compressive strength was typically associated with a lower water-to-binder ratio and a denser pore structure, which reduced the volume fraction of connected pores available for ionic transport and increased tortuosity. As a result, the apparent diffusion coefficient decreased and a lower chloride concentration profile was obtained. Meanwhile, the denser hydration product assemblage enhanced chloride binding and retention, which further reduced the driving force for free chloride transport into the interior. Therefore, high-strength concrete generally exhibited improved resistance to chloride ingress under the same exposure conditions [34].

As shown in Table 5, the effect of exposure condition on chloride transport was significant for all four pairs: C45NC0/C45DC0 (F(1,10) = 11.17, *p* = 0.00746), C50NC0/C50DC0 (F(1,10) = 9.63, *p* = 0.01119), C45NC1/C45DC1 (F(1,10) = 13.31, *p* = 0.00448), and C50NC1/C50DC1 (F(1,10) = 9.47, *p* = 0.01170). The depth effect was also highly significant in all cases (*p* < 0.001). These results confirmed that, compared with natural immersion, drying–wetting cycles significantly increased chloride transport in concrete.

Similarly, in Table 6, reinforcement configuration was used as the treatment factor to evaluate its effect on chloride concentration profiles. Significant treatment effects were observed for C45NC0/C45NC1 (F(1,10) = 60.83, *p* = 1.47 × 10^−5^), C50NC0/C50NC1 (F(1,10) = 26.67, *p* = 4.23 × 10^−4^), C45DC0/C45DC1 (F(1,10) = 31.05, *p* = 2.37 × 10^−4^), and C50DC0/C50DC1 (F(1,10) = 35.79, *p* = 1.35 × 10^−4^). The depth effect was highly significant in all cases (*p* < 0.001). These results confirmed that reinforcement configuration significantly affected the chloride concentration distribution under both NC and DC conditions.

In Table 7, compressive strength level was used as the treatment factor to evaluate its effect on chloride concentration profiles. The results for C45NC0/C50NC0 (F(1,10) = 21.20, *p* = 9.74 × 10^−4^), C45DC0/C50DC0 (F(1,10) = 32.69, *p* = 1.93 × 10^−4^), C45NC1/C50NC1 (F(1,10) = 35.14, *p* = 1.45 × 10^−4^), and C45DC1/C50DC1 (F(1,10) = 34.84, *p* = 1.51 × 10^−4^) showed that compressive strength significantly affected the chloride concentration distribution. C50 exhibited lower chloride concentration levels than C45.

## 4. Verification of Model

**Figure 9:**
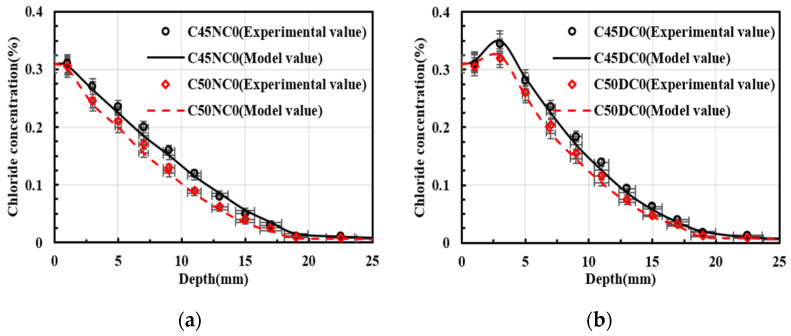

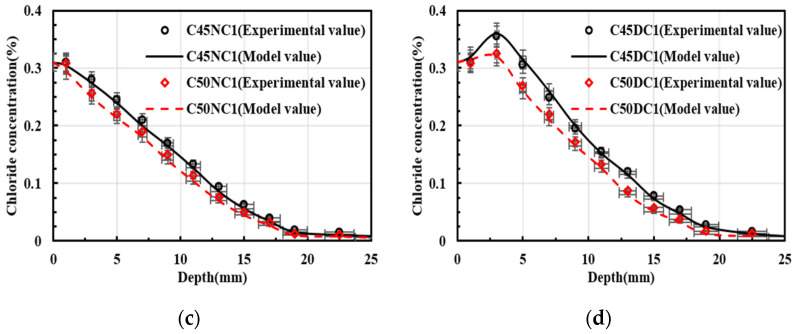
Verification of chloride concentration (**a**) No longitudinal steel, and natural immersion; (**b**) No longitudinal steel and drying–wetting cycles; (**c**) Longitudinal steel, and natural immersion; (**d**) Longitudinal steel, and drying–wetting cycles.

**Figure 10:**
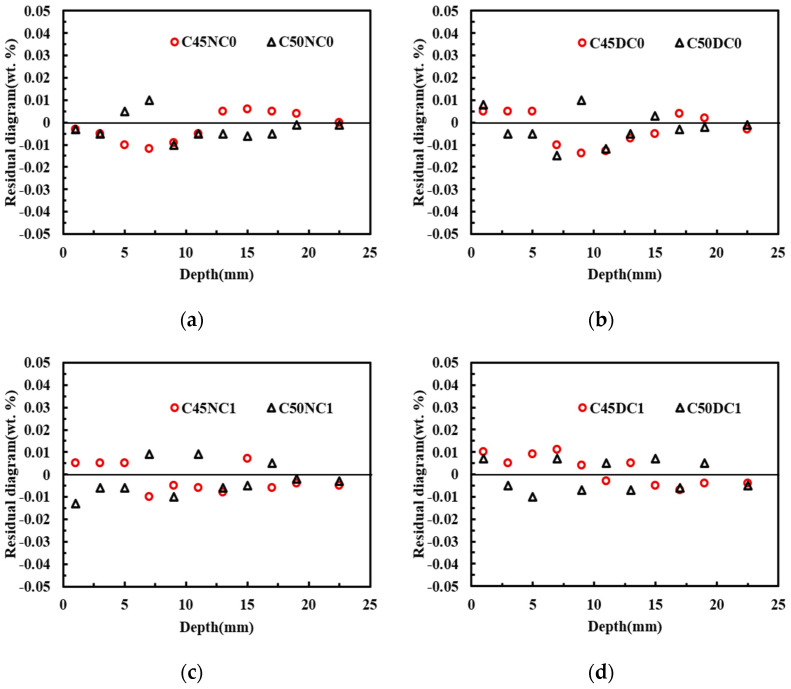
Residual distribution of chloride concentration. (**a**) C45, and no longitudinal steel; (**b**) C50, and no longitudinal steel; (**c**) C45, and longitudinal steel; (**d**) C50, and longitudinal steel.

Figure 9 compares the experimentally measured chloride concentrations with the numerical solutions. As shown in Figure 9a, the experimental results for C45NC0 agreed well with the model predictions. For C50NC0, a relatively large deviation occurred at a diffusion depth of 7 mm, with a relative error of 12.56%, whereas the discrepancies at the other depths were smaller. Similarly, in Figure 9b, the experimental values for C45DC0 were approximately evenly distributed on both sides of the predicted curve. An exception was observed at a diffusion depth of 11 mm, where the relative error between the experimental and predicted values reached 14.55%. In Figure 9c,d, good agreement was also achieved between the experimental data and the model results. Table 8 reports the mean relative error between the experimental and predicted values for each specimen. As indicated in Table 8, all mean relative errors were within 10%, which was considered acceptable [35]. Through the difference results between Table 8 and Figure 10, it can be seen that the model in this study has high accuracy.

**Figure 11:**
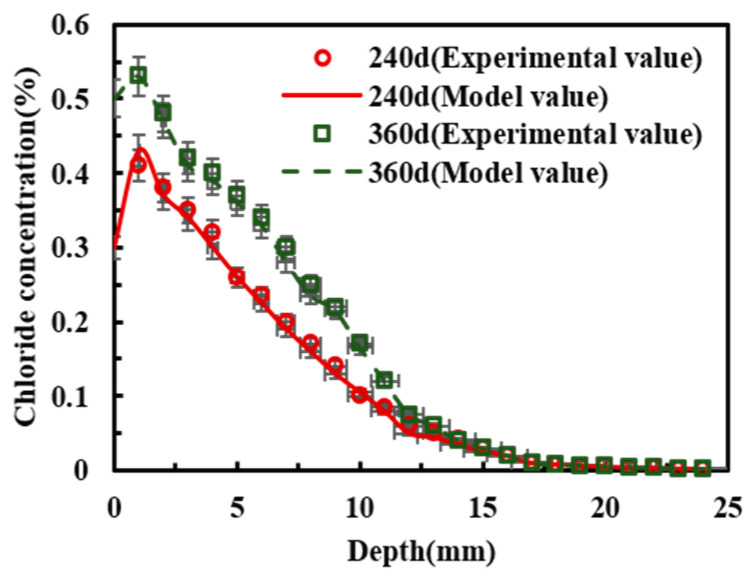
Compared with the experimental values in reference [36].

**Figure 12:**
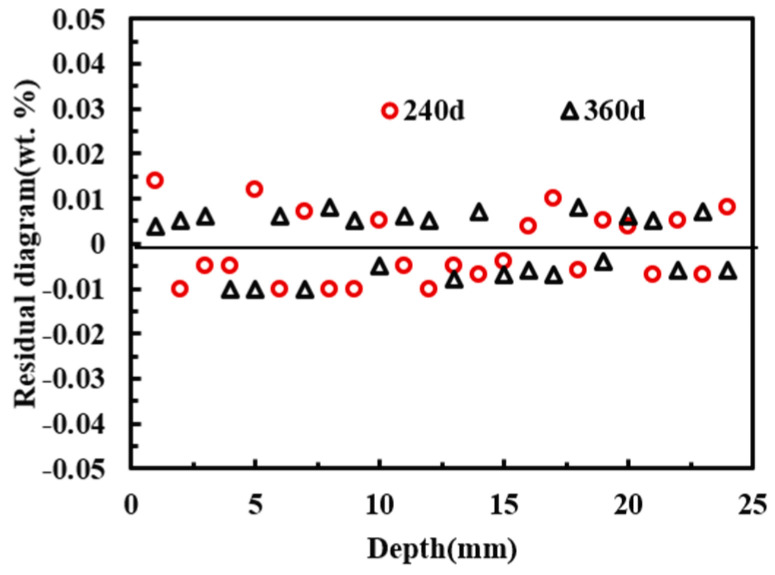
Residual distribution of chloride concentration with Xu et al. [36].

To further verify the applicability of the meso-scale model proposed in this study, the chloride exposure test data reported in reference [36] were used for model validation. The specimens had dimensions of 150 mm × 150 mm × 300 mm and incorporated four HRB335 longitudinal bars with a nominal diameter of 14 mm, with a concrete cover thickness of 25 mm. The exposure durations were 240 d and 360 d. Model development followed Section 3.1 and Section 3.2, and the associated parameters were adopted according to Table 5. As shown in Figure 11, the experimental results reported in reference [36] agreed well with the model predictions, indicating that the proposed meso-scale chloride transport model for reinforced concrete was applicable. Through the difference between Table 9 and Figure 12, it can be seen that the model in this study has high accuracy.

## 5. Sensitive Parametric Analysis

**Figure 13:**
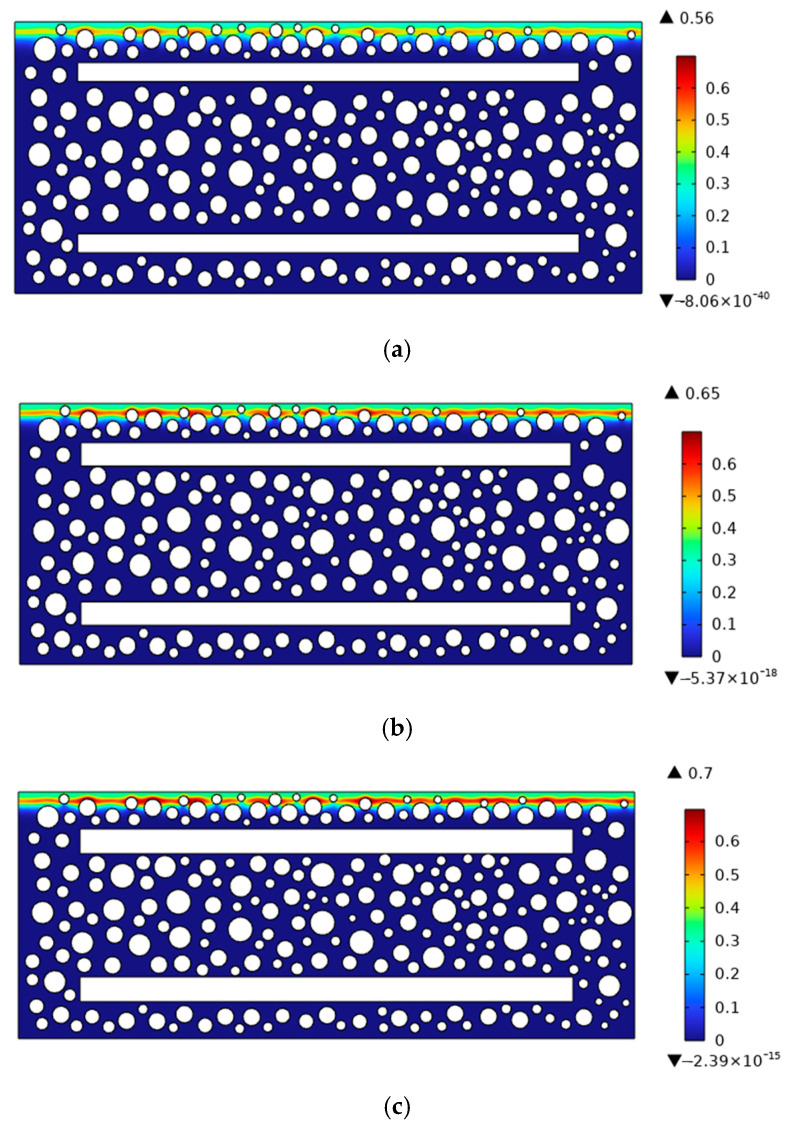
Chloride diffusion contour maps in reinforced concrete with different longitudinal reinforcement diameters. (**a**) 14 mm diameter longitudinal reinforcement; (**b**) 18 mm diameter longitudinal reinforcement; (**c**) 20 mm diameter longitudinal reinforcement.

To investigate variations in chloride transport in the vicinity of reinforcement, the experiment beam shown in Figure 1 was adopted as the reference specimen. Reinforced concrete members incorporating longitudinal bars with diameters of 14 mm, 18 mm, and 20 mm were considered for comparative analysis. The wet-to-dry duration ratio was set to 2:1, and the total exposure period was 180 days. The concrete grade was C45, and the model parameters were taken according to Table 1 and Table 5. The effect of chloride ions on the stirrups was not considered at this stage. Figure 13 presents contour maps of the chloride diffusion distribution in reinforced concrete with different longitudinal bar diameters. Under the combined effects of rebar-induced blocking and drying–wetting cycles, a substantial accumulation of chloride ions occurred around the aggregates, resulting in elevated chloride concentrations in the aggregate vicinity. Moreover, as the rebar diameter increased, not only did the peak chloride concentration rise, but the chloride concentration around the aggregates also increased more rapidly.

**Figure 14:**
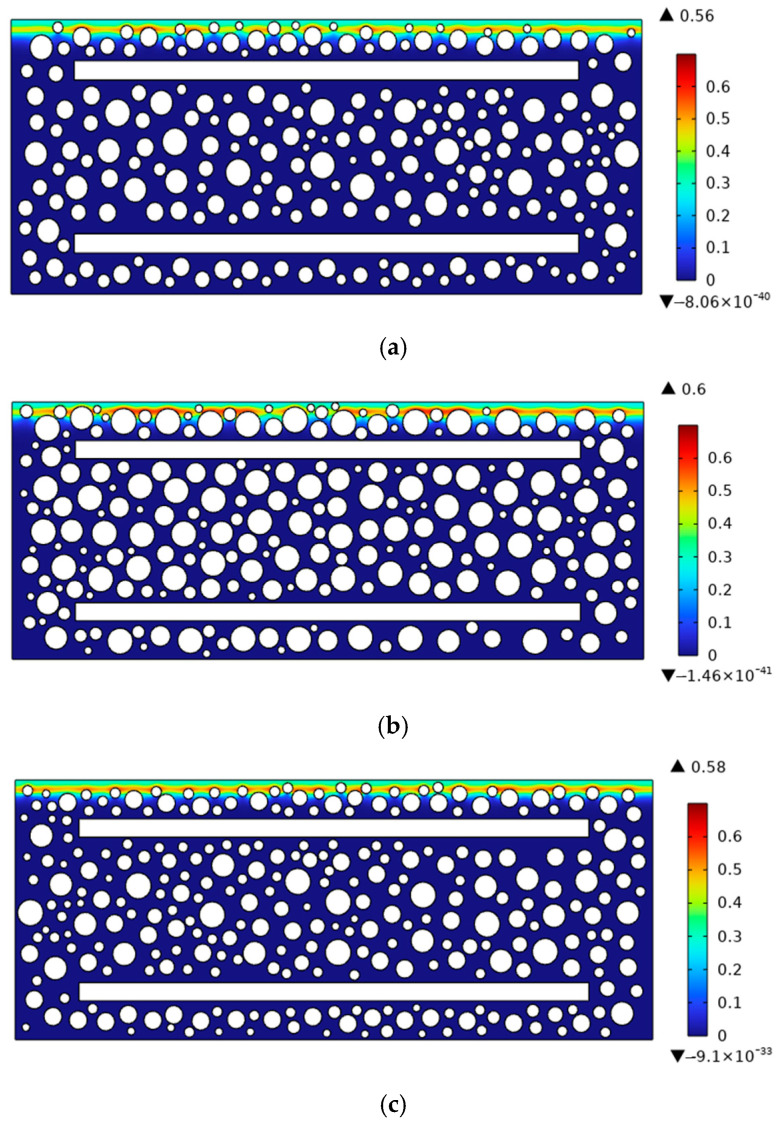
Chloride diffusion contour maps in reinforced concrete with different aggregate distributions. (**a**) This study; (**b**) Type 1; (**c**) Type 2.

**Figure 15:**
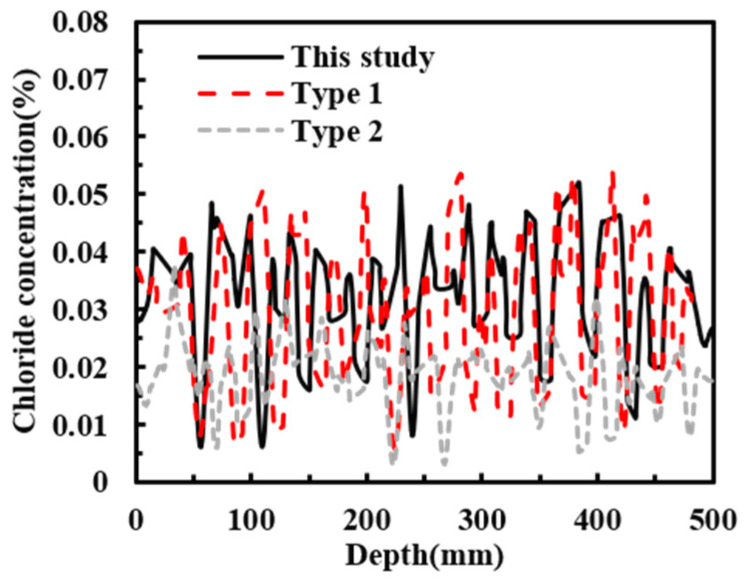
Chloride concentration of different aggregate distributions on the cross-section with diffusion depth of 20 mm.

**Figure 16:**
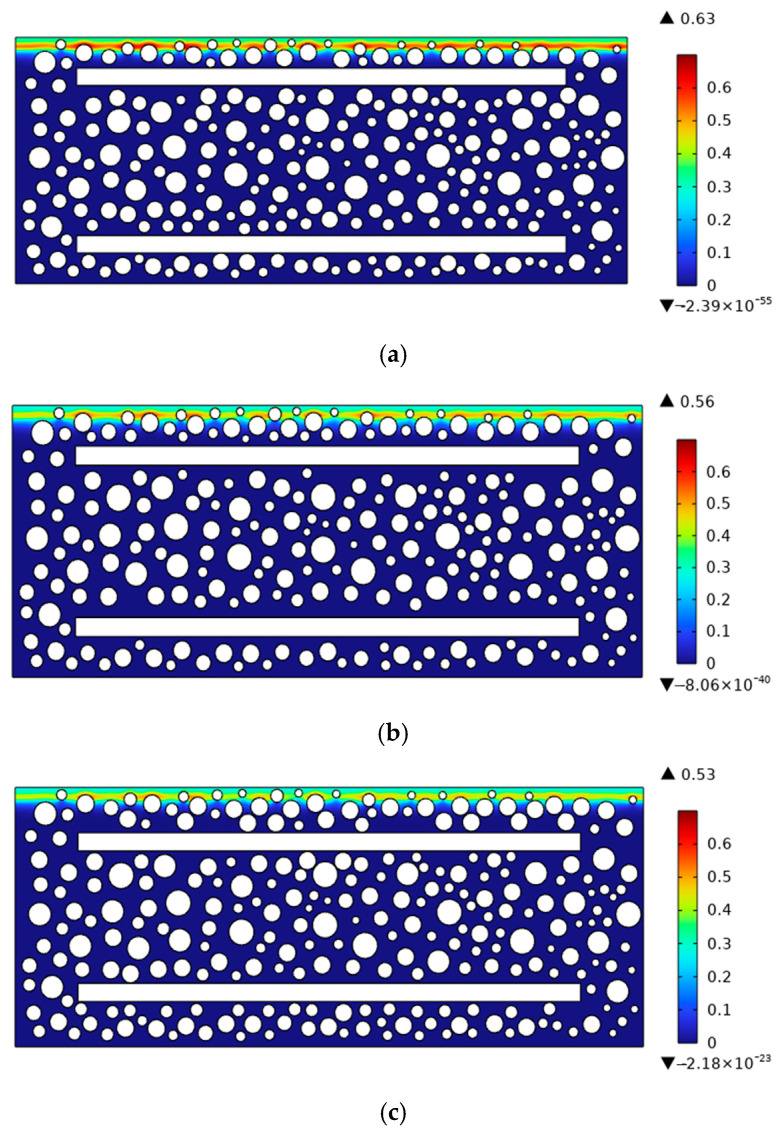
Chloride diffusion contour maps in reinforced concrete with different protective layer thicknesses. (**a**) a = 25 mm; (**b**) a = 30 mm; (**c**) a = 35 mm.

**Figure 17:**
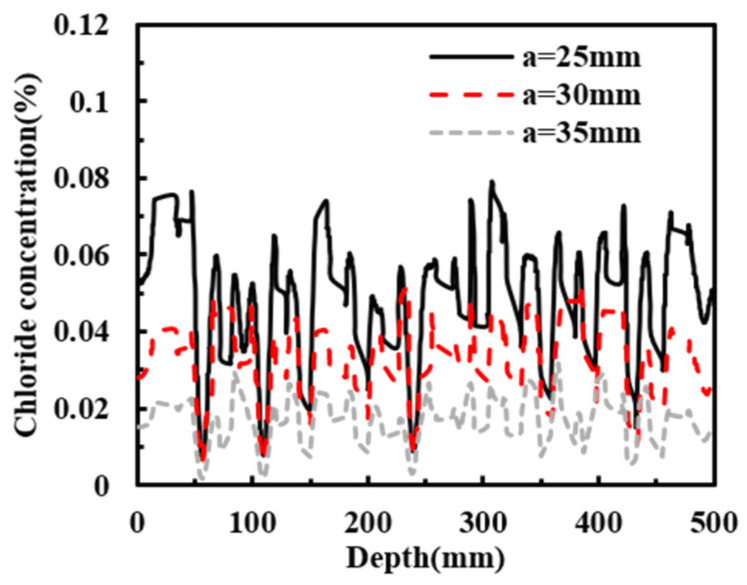
Chloride concentration of different protective layer thicknesses on the cross-section with diffusion depth of 20 mm.

To investigate the effects of aggregate arrangement and rebar position on chloride transport, the chloride distribution in reinforced concrete with an age of 180 days and a concrete cover thickness of 30 mm (a = 30 mm) was taken as the reference case. Sensitivity analyses were then carried out for three aggregate arrangements (This study, Type 1, and Type 2) and three cover thicknesses (a = 25, 30, and 35 mm). The chloride concentrations along the cross-section at a diffusion depth of 20 mm were compared and analyzed, as shown in Figure 14, Figure 15 and Figure 16. Figure 14 and Figure 15 show that the distribution of aggregates has a clear influence on chloride transport in reinforced concrete. When more coarse aggregates are located near the exposed surface, chlorides tend to accumulate more readily on their surfaces during diffusion, whereas less chloride accumulation is observed around smaller aggregates. The results further indicate that aggregate arrangement affects the chloride distribution at the same depth. As shown in Figure 16, decreasing the cover thickness enhances the blocking effect of the reinforcing bar, resulting in a higher peak chloride concentration. Consistently, Figure 17 shows that the chloride concentration for a cover thickness of 25 mm is significantly higher than that for the other cover thicknesses. These results demonstrate that the rebar position also plays an important role in chloride distribution. Overall, both aggregate distribution and rebar position affect chloride diffusion in reinforced concrete, while the influence of rebar position is more significant. To sum up, the diameter of steel bars has a great influence on chloride transport, and it cannot be ignored.

## 6. Conclusions

A series of chloride transport experiments on reinforced concrete under drying–wetting cycles was conducted to evaluate the effects of exposure condition, reinforcement-induced blocking, and concrete compressive strength on chloride transport. A meso-scale chloride diffusion model incorporating the rebar-induced blocking effect was then developed and validated. The meso-scale modeling framework was further used to investigate chloride diffusion for different longitudinal bar diameters. The following main conclusions were drawn:

1. Compared with concrete without longitudinal reinforcement, drying–wetting cycles produced a more pronounced effect on concrete incorporating longitudinal bars.
2. Drying–wetting cycles provided the strongest enhancement of chloride ingress, followed by compressive strength, and then the rebar-induced blocking effect. The blocking effect of reinforcement remained non-negligible.
3. The relative errors between the experimental measurements and the numerical solutions were small, indicating that the proposed meso-scale chloride diffusion model incorporating the rebar-induced blocking effect was applicable.
4. Under the combined effects of reinforcement-induced blocking and drying–wetting cycles, substantial chloride accumulation occurred around the aggregates, leading to elevated chloride concentrations in their vicinity. As the rebar diameter increased, the peak chloride concentration increased, and the chloride concentration around the aggregates also rose more rapidly. The influence of steel bar diameter change on chloride concentration cannot be ignored.

The present results have important implications for durability design and service-life prediction. Although the rebar-induced blocking effect is weaker than the effects of wetting–drying cycles and compressive strength, it is still non-negligible and directly affects the local chloride concentration near the reinforcement. This effect may be incorporated into engineering design through a correction to the effective chloride diffusivity, a local amplification factor for chloride concentration at the rebar depth, or an equivalent adjustment of cover efficiency. Conventional service-life models may underestimate the chloride concentration near the reinforcement when the blocking effect of steel is neglected, especially for larger rebar diameters and smaller cover thicknesses. Therefore, this effect may be introduced into corrosion initiation assessment as a local modification of chloride transport parameters. Although the present study does not provide direct code values, it provides a basis for future calibration and for incorporation into durability design provisions.

## Figures and Tables

**Table 1:** Mix ratio and related parameters of concrete.

Specimens	Water (kg/m^3^)	Cement (kg/m^3^)	Fine Aggregate (kg/m^3^)	Coarse Aggregate (kg/m^3^)
C45	180 ± 3	360 ± 4	744 ± 11	1116 ± 20
C50	180 ± 3	450 ± 3	673 ± 10	1097 ± 13

**Table 2:** Chemical compositions of cement.

Chemical Composition	SiO_2_	Al_2_O_3_	Fe_2_O_3_	CaO	MgO	SO_3_	TiO_2_	K_2_O	Na_2_O	Loss on Ignition
Mass percent (%)	20.86 ± 0.2	6.43 ± 0.1	4.76 ± 0.08	61.35 ± 0.3	1.49 ± 0.05	1.76 ± 0.06	0.25 ± 0.03	0.36 ± 0.03	0.13 ± 0.04	2.61 ± 0.11

Note: The unnormalized raw total was 99.83%, and the deviation was attributed to minor constituents and measurement uncertainty. The values were normalized and reported to two decimal places.

**Table 3:** Experiment specimen number.

Specimens	Expose the Environment	Configure the Reinforcing Bars
C45NC0	Natural immersion	-
C50NC0	Natural immersion	-
C45NC1	Natural immersion	Longitudinal steel
C50NC1	Natural immersion	Longitudinal steel
C45DC0	Drying–wetting cycles	-
C50DC0	Drying–wetting cycles	-
C45DC1	Drying–wetting cycles	Longitudinal steel
C45DC1	Drying–wetting cycles	Longitudinal steel

**Table 4:** Main parameters of calculation model.

Parameter	Value	Reference	Parameter	Value	Reference
*D*_co_w0_ (m^2^/s)	4.05 × 10^−11^	[29]	*D*_cp_ (m^2^/s)	3.4 × 10^−12^	-
*D*_co_d0_ (m^2^/s)	1.4 × 10^−10^	[29]	*s_1_*	1	[24]
*s*_c_	0.792	[24]	*s_2_*	1	[24]
*C*_s_ (%)	0.31	[24]	*s_3_*	0.45	[24]

**Table 5:** Summary of ANOVA results for NC–DC comparisons (exposure condition).

Comparison	Increase in DC vs. NC (%)	F (Treatment)	*p* (Treatment)	F (Block: Depth)	*p* (Block: Depth)
C45NC0/C45DC0	16.67	11.17	0.00746	106.22	8.62 × 10^−9^
C50NC0/C50DC0	18.78	9.63	0.01119	85.81	2.46 × 10^−8^
C45NC1/C45DC1	18.24	13.31	0.00448	91.24	1.82 × 10^−8^
C50NC1/C50DC1	15.73	9.47	0.0117	101.23	1.09 × 10^−8^

**Table 6:** Summary of ANOVA results for NC–DC comparisons (reinforcement configuration).

Comparison	Increase of “1” vs. “0” (%)	F (Treatment)	*p* (Treatment)	F (Block: Depth)	*p* (Block: Depth)
C45NC0/C45NC1	6.98	60.83	1.47 × 10^−5^	2897.1	6.16 × 10^−16^
C50NC0/C50NC1	9.58	26.67	4.23 × 10^−4^	807.82	3.62 × 10^−13^
C45DC0/C45DC1	8.42	31.05	2.37 × 10^−4^	959.23	1.54 × 10^−13^
C50DC0/C50DC1	6.77	35.79	1.35 × 10^−4^	1993.98	4.00 × 10^−15^

**Table 7:** Summary of ANOVA results for NC–DC comparisons (compressive strength level).

Comparison	Difference in C45 vs. C50 (%)	F (Treatment)	*p* (Treatment)	F (Block: Depth)	*p* (Block: Depth)
C45NC0/C50NC0	14.06	21.2	9.74 × 10^−4^	310.44	4.25 × 10^−11^
C45DC0/C50DC0	12.04	32.69	1.93 × 10^−4^	597.47	1.63 × 10^−12^
C45NC1/C50NC1	11.35	35.14	1.45 × 10^−4^	656.64	1.02 × 10^−12^
C45DC1/C50DC1	13.77	34.84	1.51 × 10^−4^	426.47	8.76 × 10^−12^

**Table 8:** Relative error between experimental value and model value.

Parameter	C45NC0	C45NC1	C45DC0	C45DC1	C50NC0	C50NC1	C50DC0	C50DC1
Relative differences (%)	1.83	3.26	4.18	1.51	6.1	2.8	5.23	2.56
RMSE (wt. %)	0.007	0.006	0.008	0.007	0.007	0.007	0.008	0.007
*p*-value	0.277	0.05	0.129	0.64	0.071	0.05	0.086	0.051

**Table 9:** Error parameters between Xu et al. [36] experimental values and model values.

Parameter	240 d	360 d
Relative differences (%)	3.611	1.270
RMSE (wt. %)	0.008	0.007
*p*-value	0.050	0.052

## Data Availability

The original contributions presented in this study are included in the article. Further inquiries can be directed to the corresponding author.
